# Reducing Red Tape’s Negative Consequences for Leaders: The Buffering Role of Autonomous Motivation

**DOI:** 10.3389/fpsyg.2021.806388

**Published:** 2022-01-14

**Authors:** Jolien Muylaert, Robin Bauwens, Mieke Audenaert, Adelien Decramer

**Affiliations:** ^1^Department of Marketing, Innovation, and Organisation, Ghent University, Ghent, Belgium; ^2^Department of Human Resource Studies, Tilburg University, Tilburg, Netherlands

**Keywords:** administrative burden, autonomous motivation, discretionary room, elderly care homes, head nurses, job satisfaction, red tape

## Abstract

In a context where the amount of red tape in healthcare organizations continues to rise, head nurses’ job satisfaction is constantly under pressure. By building on the Job Demands-Resources model, we developed a theoretical model investigating the relationship between red tape and job satisfaction. By investigating the mediating role of discretionary room and the moderating role of autonomous motivation in this relationship, this study does not only aim to provide additional knowledge regarding the underlying mechanisms in this relationship, but also to increase our understanding of how this suffering at work can be mitigated. Our conditional process analyses (*N* = 277 head nurses) indicate that red tape undermines head nurses’ job satisfaction and that discretionary room acts as an underlying mechanism in this process. By revealing the mediating role of discretionary room, this study advances our understanding of the risks originating from red tape for leaders. Furthermore, our findings also indicate that autonomous motivation mitigates the negative relation between red tape and discretionary room and between red tape and job satisfaction. As autonomous motivation turns out to be an important protection mechanism against the negative consequences of red tape, organizations should put extra effort into stimulating the autonomous motivation of their leaders. When organizations make sure that their leaders’ job designs and work environments meet the need for autonomy, competence, and relatedness, leaders will become more autonomously motivated, which will buffer the negative impact of red tape.

## Introduction

Red tape is an increasing problem in many organizations around the world (e.g., Kaufmann et al., 2019). When rules, regulations, and procedures entail a compliance burden, but lack functionality, they can be categorized as red tape (Bozeman and Feeney, 2014). Healthcare workers in particular are complaining about the increasing levels of red tape they are facing in their job (e.g., Steijn and Van der Voet, 2019). This is alarming as past research demonstrates that red tape is negatively related to numerous outcomes (George et al., 2021). This study explores how red tape affects head nurses’ job satisfaction, by investigating the mediating role of discretionary room and the moderating role of autonomous motivations. With this focus, this study does not only address a call for research to investigate the underlying mechanisms that explain why workers are affected by red tape in a certain way (George et al., 2021), particularly leaders, but also explores how red tape induced suffering at work can be mitigated. While the previous research efforts have focused on demonstrating the negative relation between red tape and job satisfaction, this study provides a coping strategy that helps leaders deal with this work-related stressor (van Dorssen-Boog et al., 2021).

By building on the Job Demands-Resources (JD-R) model (Bakker and Demerouti, 2007), we focus on red tape as a hindering job demand with negative consequences for head nurses’ job satisfaction. Job satisfaction can be defined as “a pleasurable or positive emotional state resulting from the appraisal of one’s job or job experience” (Locke, 1976, p. 1,300). Studying job satisfaction is highly relevant because it is directly related to numerous important outcomes, such as job performance (e.g., Willem et al., 2007), workers’ health (e.g., Thielgen et al., 2015), and turnover intentions (e.g., Lu et al., 2019). Importantly, specifically head nurses’ job satisfaction is also crucial given its positive association with patient satisfaction, and quality and safety of patient care (e.g., Tzeng and Ketefian, 2002; Kvist et al., 2014). However, despite its importance, head nurses’ job satisfaction is continuously challenged by multiple phenomena. For example, there is an acute lack of head nurses, which increases the workload for those in this particular position (De Broeck, 2011; Zhang et al., 2020). At the same time, head nurses are confronted with an aging population and rising patient expectations (Kowalski et al., 2010; van Oostveen et al., 2015). Given these concerns, it is important to obtain additional knowledge about which variables affect head nurses’ job satisfaction. Accordingly, in order to obtain more insights into the underlying mechanisms explaining why red tape affects head nurses’ job satisfaction in a certain way, this study explores the mediating role of discretionary room. Discretionary room refers to “the degree of freedom and authority managers have to decide what to do” (Knies and Leisink, 2014, p. 118). As line managers, head nurses are responsible for managing a team of nurses. In order to perform this task, head nurses must experience sufficient discretionary room (Rutz et al., 2017). However, in this study, we argue that head nurses’ discretionary room is limited by the presence of red tape, which in turn will lower head nurses’ job satisfaction.

Although red tape should be eliminated wherever possible, it might not be feasible for organizations to remove externally imposed regulations (Walker and Brewer, 2009; Moynihan et al., 2012). If the elimination of red tape is impossible, head nurses would benefit from the development of coping strategies to help them deal with the presence of red tape in their job (van Dorssen-Boog et al., 2021). One such coping strategy that could be particularly relevant, is the extent to which head nurses are autonomously motivated. Autonomously motivated head nurses engage in their job out of free will, are interested in their work, and take pleasure in performing their tasks (Ryan and Deci, 2002; Gagné and Deci, 2005). Consequently, this study will investigate whether autonomously motivated head nurses are less negatively affected by red tape, as this form of motivation might help them cope with the presence of red tape. If our results confirm that autonomous motivation does indeed mitigate the negative relationship between red tape and discretionary room and/or job satisfaction, interventions with the purpose of increasing head nurses’ autonomous motivation would help them to deal with the presence of this hindering demand. Since past research shows that organizations can foster the autonomous motivation of their employees and leaders (Fernet et al., 2021), organizations should put extra effort into stimulating autonomous motivation. When directors make sure that job designs and work environments meet the need for autonomy, competence, and relatedness, their workers, including those with managerial responsibilities, will become more autonomously motivated (Stone et al., 2009; De Cooman et al., 2013; Li et al., 2021), which, as argued in this study, will buffer the negative impact of red tape.

In testing our theoretical model, we aim for a two-fold contribution. First of all, more research is needed to reveal the *underlying mechanisms* that explain why employees’ (here i.e., leaders’) attitudes and behaviors are affected by red tape in a certain way (Quratulain and Khan, 2015; Kaufmann et al., 2019; George et al., 2021). By exploring the mediating role of discretionary room, this study will advance our understanding of the risks that originate from red tape. More specifically, this study adds to the literature on the negative relationship between red tape and job satisfaction (e.g., Steijn and Van der Voet, 2019), as there is a lack of research investigating possible mediators between red tape and behavioral outcomes (Quratulain and Khan, 2015; George et al., 2021). While addressing this gap, this study is also innovative by focusing on a mediator that is of particular importance in leadership roles (i.e., discretionary room; Knies and Leisink, 2014, 2018). Secondly, while previous literature focused on demonstrating the negative relation between red tape and job satisfaction, the present study explores a way to actually mitigate red tape’s negative consequences. By focusing on autonomous motivation as a personal resource, this study contributes to research regarding the impact of personal resources, when being confronted with red tape (Bakker and Demerouti, 2018). By doing so, this study contributes to our understanding of how leaders can cope with the negative effects of red tape. Additionally, it is important to note that the implications of our findings go beyond the healthcare context, as past research shows that the negative impact of red tape is quite stable across different contexts (George et al., 2021).

## Background and Hypotheses

### Red Tape and Job Satisfaction

Bozeman (2000) defined red tape as “rules, regulations, and procedures that remain in force and entail a compliance burden, but do not advance the legitimate purposes the rules were intended to serve” (p. 12). Following this definition, rules, regulations, and procedures have to score high on two characteristics before they can be categorized as red tape. The first characteristic is compliance burden, which refers to the effort and time it takes to comply with a certain rule (van Loon, 2017). However, having a high compliance burden alone is not sufficient to declare a rule ‘red tape,’ since some burdensome rules are worth the trouble, for example, hospital safety guidelines (van Loon et al., 2016). Therefore, a second characteristic, namely lack of functionality, is necessary to make a distinction between red tape and burdensome rules in general (van Loon et al., 2016). Lack of functionality refers to the extent to which a rule serves the purpose that it is intended to regulate (van Loon, 2017). Nevertheless, it is important to note that this characteristic should be interpreted as the *perceived* degree to which rules, regulations, and procedures lack functionality rather than as having absolutely no functionality at all (Bozeman, 2012; van Loon et al., 2016).

Red tape in healthcare organizations originates from a rising focus on quality assurance and performance indicators, in combination with rising accountability expectations imposed by governments and organizations alike (Savaya et al., 2006; Bail et al., 2009; Borst et al., 2020; George et al., 2021). Contemporary healthcare organizations are required to provide data on quality, safety, performance, and efficiency, resulting in growing levels of red tape perceptions (Scott and Pandey, 2005; Lega and Sartirana, 2016). While the purpose of this increasing amount of paperwork is to benefit patient safety and performance in general, it often results in the opposite, since this paperwork is generally experienced as an extra burden on a staff that is already overworked (Hansen et al., 2003). Moreover, red tape also reduces the time spent with patients and hinders head nurses, and healthcare workers in general, to handle complex cases well (Brodkin, 2012; van Oostveen et al., 2015; van Loon, 2017).

By building on the JD-R model, we expect red tape to be negatively related to job satisfaction. The JD-R model presumes that all job characteristics can be divided into job demands and job resources (Bakker and Demerouti, 2007, 2018). Job demands refer to the aspects of a job that cost energy, whereas job resources refer to the aspects of work that help people to deal with job demands, help them to satisfy their basic psychological needs, and help them to achieve organizational goals (Bakker, 2015). Moreover, people can also possess personal resources. Personal resources are “aspects of the self that are generally linked to resiliency and refer to individuals’ sense of their ability to control and impact upon their environment successfully” (Xanthopoulou et al., 2007, pp. 123–124). Additionally, job demands can be divided into challenging and hindering job demands. Challenging job demands, such as complex tasks, can motivate people to perform well (Quratulain and Khan, 2015; Bakker and Demerouti, 2018). Hindering job demands, on the other hand, refer to working conditions that involve undesirable or excessive constraints that inhibit individuals’ abilities to attain goals and undermine their performance.

The JD-R model proposes that hindering job demands have a negative effect on well-being (Steijn and Van der Voet, 2019; Shin et al., 2020). Well-being can be defined as the overall quality of an individual’s experience and functioning at work, and can be understood as a three-dimensional model, consisting of a psychological, a physical, and a social dimension (Grant et al., 2007). Consequently, as job satisfaction is an indicator of psychological well-being, job satisfaction is positively related to well-being (Grant et al., 2007).

Since red tape is an aspect of work that costs energy and inhibits individuals’ abilities to achieve goals (Quratulain and Khan, 2015), red tape is by its nature a hindering job demand (e.g., Bakker and Demerouti, 2018; Wang and Guan, 2018), which will negatively affect job satisfaction (Steijn and Van der Voet, 2019). More specifically, two recent red tape meta-analyses revealed that red tape enhances different forms of alienation, such as feelings of powerlessness and meaninglessness, which, in turn, are harmful to numerous well-being outcomes, including job satisfaction (Blom et al., 2021; George et al., 2021). Additionally, and more specifically to our research context, although red tape is a hindrance for all types of workers, it can be especially impeding for healthcare personnel (Borst, 2018). Healthcare workers often experience their work as a calling and want to focus their time on taking care of patients (Tuckett et al., 2015; Borst, 2018). They experience time spent on paperwork as lost time, since they can no longer spend it on healing patients (Borst, 2018). For them, the human aspect of their job is far more important than the administrative aspect (Tuckett et al., 2015). Seeing patients well taken care of is what brings them job satisfaction, administrative burden brings the opposite (Nolan et al., 2007; Tuckett et al., 2015).

In sum, since the JD-R model states that hindering job demands negatively influence well-being (Kahn, 1990; Schaufeli, 2015; Borst et al., 2019), we expect that head nurses will have a lower job satisfaction when they perceive a great amount of red tape. In support of this reasoning, previous research found that red tape is indeed negatively associated with job satisfaction (Borst, 2018; Steijn and Van der Voet, 2019; Blom et al., 2021). Given these arguments, we hypothesize that:

Hypothesis 1: Head nurses’ red tape perceptions are negatively related to their job satisfaction.

### The Mediating Role of Head Nurses’ Discretionary Room

Only recently, scholars started to build on the JD-R model in order to explain the negative relation between red tape and well-being outcomes (Blom et al., 2021). Underlying this literature is the assumption that red tape reduces individuals’ autonomy, which alienates them from their work, which, in turn, is detrimental to their well-being, including their job satisfaction (Borst et al., 2019; Blom et al., 2021; George et al., 2021). Moreover, as a more recent, and extended, version of the JD-R model states that job demands do not occur in isolation from other job characteristics, researchers claim that the presence of red tape also negatively affects other job characteristics (Burton and Van den Broek, 2009; Bakker and Demerouti, 2017, 2018; Steijn and Van der Voet, 2019). Consequently, we expect discretionary room to mediate the relationship between red tape and job satisfaction. Discretionary room relates to managers’ freedom in adapting the job, making tailor-made arrangements for their staff, and making use of career opportunities (Knies and Leisink, 2014).

Past research shows that organizational characteristics affect the degree of managerial discretion leaders experience within their organization (Hambrick and Finkelstein, 1987; Knies and Leisink, 2018). Accordingly, we argue that red tape will limit head nurses’ discretionary room, as the presence of rules, regulations, and procedures that comprise a compliance burden, but have no functionality, will inhibit their freedom and authority to decide what to do (Bozeman, 1993; Knies and Leisink, 2014; Steijn and Van der Voet, 2019). When leaders experience insufficient room to shape individual arrangements with their employees, they will perceive less managerial autonomy (Knies and Leisink, 2014, 2018). Since autonomy is an important condition for healthcare workers’ job satisfaction (Hackman and Oldham, 1976; Finn, 2001; Dysvik and Kuvaas, 2011; Steijn and Van der Voet, 2019), we argue that lower levels of discretionary room will result in a declining job satisfaction.

In line with the above reasoning, several studies suggest that rules and regulations limit managerial discretion (Boyne et al., 1999; Truss, 2008; Rainey, 2009; Brown, 2010; Knies and Leisink, 2018). In turn, a study by Van der Voet and Van de Walle (2018), as well as a study by Brunetto et al. (2017), shows that managerial autonomy positively predicts job satisfaction. Taken together, these arguments suggest that head nurses’ red tape perceptions are negatively associated with their discretionary room, ultimately decreasing their job satisfaction. Consequently, we hypothesize:

Hypothesis 2: Head nurses’ discretionary room partially mediates the negative relationship between red tape and job satisfaction. More specifically, red tape is negatively related to discretionary room (H2a), which in turn is positively related to job satisfaction (H2b).

### The Moderating Role of Autonomous Motivation

Next, we identify head nurses’ autonomous motivation as a potential moderating condition, attenuating the relationship between red tape and discretionary room and between red tape and job satisfaction. Autonomous motivation refers to “the full endorsement of one’s own activities, as these are in concordance with personal goals, needs, interests, and values” (van Dorssen-Boog et al., 2021, p. 260).

The JD-R model states that not everyone is equally affected by the hindering aspects of their job, since personal resources can act as a buffer against the negative impact of hindering job demands on well-being (Trépanier et al., 2013; Bakker, 2015). More specifically, personal resources can help people to actively approach their hindering job demands and can help them to deal with these demands in an effective way (Bakker and Demerouti, 2018). Autonomous motivation can be categorized as such a personal resource (Trépanier et al., 2013).

We argue that autonomously motivated head nurses are better equipped to handle red tape since their autonomous motivation helps them to arm themselves with the resources it takes to cope with red tape (Trépanier et al., 2013; Bakker and Demerouti, 2018). Applied to the impact on job satisfaction, we argue that head nurses who experience high levels of autonomous motivation will initiate personal initiatives to modify their work environment and mobilize their job resources in order to remain satisfied in their job, while being confronted with high levels of red tape (Bakker, 2015; Steijn and Van der Voet, 2019). Moreover, past literature revealed that motivated individuals are more involved in their jobs, demonstrate greater goal attainment, are more persistent, and are more self-driven (Gagné and Deci, 2005; Kuvaas and Dysvik, 2010; Dysvik and Kuvaas, 2011; Kusurkar, 2019). Applied to the impact on discretionary room, these observations suggest that autonomously motivated individuals will respond less negatively to the presence of red tape, because their goal-driven orientation makes them eager to find, and apply, discretion in any possible domain of their job, regardless of the level of red tape (Dysvik and Kuvaas, 2011). Consequently, we argue that when head nurses are confronted with red tape in their job, while at the same time having high levels of autonomous motivation, that they will be persistent to modify their work environment in order to obtain more discretionary room (Bakker, 2015).

In support of the above reasoning, past research suggests that nurses who possess many personal resources are better able to cope with bureaucracy (Bakker and Demerouti, 2018). Additionally, a study by Trépanier et al. (2013) identified autonomous motivation as an important personal resource, as the results of their study revealed that the well-being of highly autonomously motivated employees was less negatively affected by the presence of different job demands, as compared to the well-being of those employees who were less autonomously motivated. Thus, we predict:

Hypothesis 3a: The relationship between head nurses’ red tape perceptions and their job satisfaction is moderated by autonomous motivation, in such a way that the negative relationship between red tape and job satisfaction is weaker when autonomous motivation is high.

Hypothesis 3b: The relationship between head nurses’ red tape perceptions and their discretionary room is moderated by autonomous motivation, in such a way that the negative relationship between red tape and discretionary room is weaker when autonomous motivation is high.

Finally, considering that discretionary room is hypothesized to mediate the relationship between red tape and job satisfaction (Hypothesis 2) and that the red tape-discretionary room relationship is predicted to be dependent on autonomous motivation (Hypothesis 3b), we expect autonomous motivation to act as a moderator in determining the strength of the indirect relation of red tape with job satisfaction via discretionary room. Such that this mediated relationship is expected to be weaker when autonomous motivation is high. Consequently, moderated mediation is expected. Stated formally:

Hypothesis 4: Head nurses’ autonomous motivation will moderate the mediated relationship between red tape and job satisfaction via discretionary room, such that the mediated relationship will be weaker when autonomous motivation is higher.

## Materials and Methods

### Participants and Procedure

We recruited a convenience sample of head nurses in elderly care homes in Flanders. We only included head nurses from public elderly care homes, as these types of homes are especially susceptible to mounting rules and regulations (Knies et al., 2018).

To collect the data, structured paper-and-pencil questionnaires were distributed in elderly care homes in October 2017. The directors of the nursing homes were informed about the aim and scope of the research. To ensure anonymity, responses were collected in sealed envelopes. Perceptual data from surveys is well suited to study red tape, discretionary room, autonomous motivation, and job satisfaction, as ‘private experiences’ for which no other data is available (Conway and Lance, 2010). However, this kind of data is also susceptible to common source bias (CSB). Therefore, we took into account a series of provisions before and after the data collection to mitigate such bias (Podsakoff et al., 2012; George and Pandey, 2017). First of all, we only included measures with established psychometric properties. Secondly, we stressed respondents’ anonymity, as well as the importance of their personal opinions and voluntary participation. Thirdly, we induced a psychological time lag by isolating independent and dependent variables in different questionnaire chapters. Finally, the severity of CSB was established through a one-factor model.

We contacted the directors of all 293 public elderly care homes in Flanders, which mostly employ 2–3 head nurses. We received responses from 108 public elderly care homes (36.86%), to which we send out 330 questionnaires. These were returned by 277 head nurses (83.94%). The average head nurse was 45 years old (range 22–77, *SD* = 9.67) and had 11.04 years of experience in this role (range 0–35, *SD* = 8.06). The majority of head nurses were female (79.78%) and were responsible for about 19.24 nurses (range 2–50, *SD* = 8.89).

### Measures

All measures were administered in Dutch on a seven-point Likert scale (1 = totally disagree, 7 = totally agree). Back translation was used where Dutch items were unavailable.

*Red tape* is not only determined by objective organizational conditions, but also depends on individuals’ subjective perceptions (Rainey et al., 1995; Scott and Pandey, 2005; Kaufmann et al., 2019). Accordingly, we used a perceptual measure, being van Loon et al.’s (2016) nine-item scale (e.g., ‘The rules I have to adhere to take a lot of time to comply with’). Cronbach’s alpha was 0.81.

*Discretionary room* was measured using Knies and Leisink’s (2014) five-item scale (e.g., ‘I experience sufficient room to make individual arrangements with an employee about doing their job’). Cronbach’s alpha was 0.81.

*Job satisfaction* was gauged by a single item: ‘All things considered, I am satisfied with my job.’ Past research which specifically focused on investigating the reliability and validity of a single-item measure of job satisfaction, including a meta-analysis, suggests that a single-item approach is a reliable way to measure job satisfaction (e.g., Wanous et al., 1997; Nagy, 2002; Dolbier et al., 2005). Consequently, this single-item measure of job satisfaction has often been used by researchers in the past (e.g., Steijn, 2004; Vermeeren et al., 2011).

*Autonomous motivation* was assessed using Gagné et al.’s (2015) six-item scale (e.g., ‘I would put effort into the job because the work I do is interesting’). Cronbach’s alpha was 0.86.

In line with Bernerth and Aguinis’s (2016) guidelines, we added *control variables* that altered perceptions of discretionary room and job satisfaction in past research, like gender (e.g., Zou, 2015), age (e.g., Piosik et al., 2019), and span of control (e.g., Davison, 2003; Lee and Cummings, 2008).

### Data Analysis

Intraclass correlation coefficients (ICCs) for red tape (ICC1 = 0.07; ICC2 = 0.16), autonomous motivation (ICC1 = 0.08; ICC2 = 0.18), discretionary room (ICC1 = 0.18; ICC2 = 0.35), and job satisfaction (ICC1 = −0.02; ICC2 = −0.05) indicated little common variance of head nurses (level 1) clustered within elderly care homes (level 2) (Fleiss, 1986; LeBreton and Senter, 2008). Therefore, we analyzed a 1–1–1 mediation with a level-1 moderator, following two steps. First, we tested the latent variable structure of the model (measurement model) by means of confirmatory factor analysis (CFA). Since our dependent variable is an endogenous categorical variable, we adopted diagonally weighted least squares estimation (DWLS), which is more robust than maximum likelihood and takes into account the categorical nature of the survey items (Li, 2016). In comparing alternative measurement and structural models, we used prominent fit indices and thresholds, like a comparative fit index (CFI) and Tucker-Lewis index (TLI) close to 0.90, as well as a mean square error of approximation (RMSEA) and standardized root mean square residual (SRMR) close to 0.08 and 0.07, respectively (Hair et al., 2013). Second, we tested our hypotheses using conditional process analysis with the PROCESS macro for SPSS developed by Hayes (2013) (SPSS Version 27.0 and PROCESS macro version 4.0). This software calculates bootstrapped 95% confidence intervals (CI) for the (conditional) direct and indirect effects. The advantage of this approach is that it presents a more stringent way to calculate (conditional) indirect effects, given potential non-normality (MacKinnon et al., 2004). Model 4 in the PROCESS macro allowed us to test the effect of red tape on job satisfaction (Hypothesis 1), as well as the mediation effect through discretionary room (Hypothesis 2) (Hayes and Scharkow, 2013). Additionally, model 8 in the PROCESS macro allowed us to test the moderating effects of autonomous motivation (Hypothesis 3) and also enabled us to investigate the potential presence of moderated mediation (Hypothesis 4) through estimating conditional indirect effects and the index of moderated mediation. Prior to any analyses, the interaction variables were mean-centered (Cohen et al., 2003).

## Results

### Preliminary Analyses

Confirmatory factor analysis was performed to test our measurement model. An overview of these models and fit indices can be found in Table 1. The hypothesized model with 21 items on four factors fits the data relatively well (χ^2^ = 327.17, df = 182, CFI = 0.96, TLI = 0.95, RMSEA = 0.06, SRMR = 0.08). All items loaded sufficiently on their factors. Furthermore, the one-factor model showed a significantly lower fit to the data (Δχ^2^ = 789.52, Δdf = 7, *p* < 0.001). Accordingly, we can endorse the convergent and discriminant validity of the measurement model and we can conclude that significant CSB was absent. Additionally, we investigated the potential presence of multicollinearity. Our variance inflation factors (VIFs) ranged between 1.01 and 1.25, which indicates that multicollinearity is not a problem (Hair et al., 2013).

**TABLE 1:** Models and fit indices.

	χ^2^	df	CFI	TLI	RMSEA	SRMR
**Measurement models**						
Hypothesized four-factor model	327.17	182	0.96	0.95	0.06	0.08
One-factor model (CSB)	1116.69	189	0.72	0.69	0.14	0.16
**Structural model**						
Partial moderated mediation	394.42	258	0.96	0.95	0.05	0.08

*CFI, comparative fit index; TLI, Tucker–Lewis index; RMSEA, root mean square error of approximation; SRMR, standardized root mean square residual; CSB, common source bias.*

Table 2 reports the descriptive statistics and Pearson correlation coefficients of all variables that were included in our hypothesized model. The correlation coefficients indicate that age was associated with higher job satisfaction. Gender and span of control showed no significant relations with other variables. In line with the hypotheses, red tape correlated negatively with autonomous motivation, discretionary room, and job satisfaction. Additionally, autonomous motivation showed a positive association with job satisfaction. Lastly, discretionary room and job satisfaction were positively related.

**TABLE 2:** Descriptive statistics and correlations.

		Mean	*SD*	1	2	3	4	5	6
(1)	Age (in years)	45.38	9.69	–					
(2)	Gender (1 = female)	0.80	0.40	0.02	–				
(3)	Span of control	19.24	8.89	0.08	–0.01	–			
(4)	Red tape	3.29	0.74	0.04	–0.01	0.05	–		
(5)	Autonomous motivation	5.98	0.68	0.07	0.08	–0.02	−0.34***	–	
(6)	Discretionary room	4.76	0.99	–0.03	0.02	0.08	−0.30***	0.09	–
(7)	Job satisfaction	5.95	0.87	0.14*	0.09	–0.05	−0.50***	0.52***	0.28***

*p < 0.05, ***p < 0.001.

### Hypothesis Testing

**FIGURE 1:**
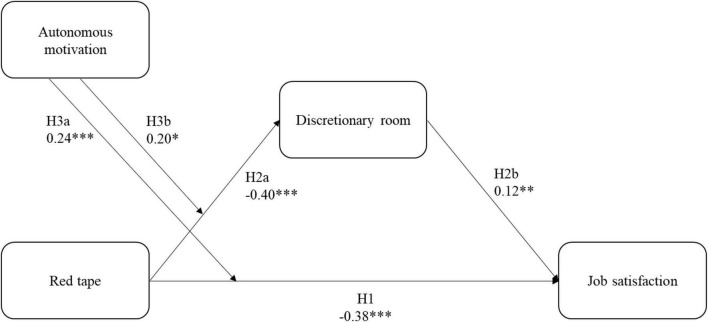
Results of the hierarchical regression analyses of the moderated mediation model. Participants’ age, gender, and span of control are included as control variables. *N* = 247; *p < 0.05, **p < 0.01, ***p < 0.001.

Table 3 reports the effect sizes of our hypothesized model. This table consists of five different models, allowing us to test our different hypotheses. To facilitate the interpretation of these models, the table clearly states which model tests which hypothesis. Also, the dependent variable of every model is clearly stated in this table. A visualization of our model is provided in Figure 1. The control variables age, gender, and span of control were included in all the analyses. Only the relation between age and job satisfaction turned out to be significant (*B* = 0.01, *p* < 0.01).

**TABLE 3:** Regression results.

Model	Model 1 (H1)		Model 2 (H2a)		Model 3 (H2b)		Model 4 (H3b)		Model 5 (H3a)	
Dependent variable	Job satisfaction		Discretionary room		Job satisfaction		Discretionary room		Job satisfaction	
	*B*	*SE*	*B*	*SE*	*B*	*SE*	*B*	*SE*	*B*	*SE*
Constant	7.21***	0.33	6.03***	0.42	6.26***	0.45	4.71***	0.33	4.85***	0.32
Age (in years)	0.01**	0.01	0.00	0.01	0.01**	0.00	0.00	0.01	0.01*	0.00
Gender (1 = female)	0.18	0.12	0.02	0.15	0.18	0.12	0.04	0.15	0.15	0.10
Span of control	0.00	0.01	0.01	0.01	0.00	0.01	0.01	0.01	0.00	0.00
Red tape	−0.60***	0.06	−0.42***	0.08	−0.53***	0.07	−0.40***	0.09	−0.38***	0.06
Autonomous motivation	–	–	–	–	–	–	0.00	0.10	0.42***	0.07
Discretionary room	–	–	–	–	0.16**	0.05	–	–	0.12**	0.05
Red tape × autonomous motivation	–	–	–	–	–	–	0.20*	0.10	0.24***	0.07
*F*-value	23.93***		7.10***		21.76***		5.56***		29.34***	
*R* ^2^	0.28		0.11		0.31		0.12		0.46	

N = 247; *p < 0.05, **p < 0.01, ***p < 0.001.

*χ^2^ = 394.42, df = 258, CFI = 0.96, TLI = 0.95, RMSEA = 0.05, SRMR = 0.08.*

Models 1, 2, and 3 in Table 3 allow us to test our first two hypotheses. Model 1 shows the total effect of red tape on job satisfaction. Model 2 shows the main effect of red tape on discretionary room, whereas model 3 shows us the main effects of red tape and discretionary room on job satisfaction. Supporting Hypothesis 1, our analysis demonstrates that red tape is indeed negatively associated with job satisfaction (total effect of red tape on job satisfaction: *B* = −0.60, *p* < 0.001). Supporting Hypothesis 2a, red tape showed a negative relation with discretionary room (*B* = −0.42, *p* < 0.001). In turn, discretionary room had a positive relation with job satisfaction (*B* = 0.16, *p* < 0.01), supporting Hypothesis 2b. Bootstrapping in 10,000 samples revealed a significant negative indirect effect for the relation red tape and job satisfaction, mediated by discretionary room (*B* = −0.07; CI: −0.13, −0.02, at a 95% CI), supporting Hypothesis 2. However, the relation between red tape and job satisfaction remained significant after adding discretionary room as a mediator (*B* = −0.53, *p* < 0.001), indicating, as we hypothesized, a partial mediation.

**FIGURE 2:**
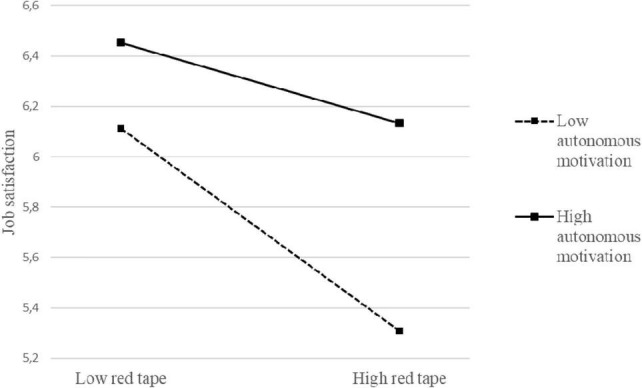
Interaction between red tape and autonomous motivation in predicting job satisfaction.

**FIGURE 3:**
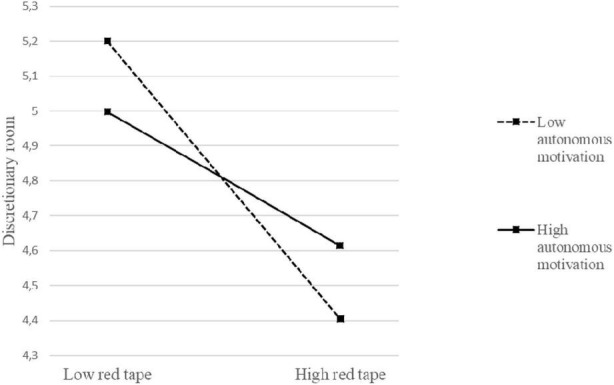
Interaction between red tape and autonomous motivation in predicting discretionary room.

Models 4 and 5 in Table 3 allow us to test Hypotheses 3 and 4. Model 4 shows the main effects of red tape, autonomous motivation, and the interaction between these variables on discretionary room. Model 5, on the other hand, shows the main effects of red tape, autonomous motivation, the interaction between these variables, and discretionary room on job satisfaction. The interaction between red tape and autonomous motivation was significantly related to job satisfaction (*B* = 0.24, *p* < 0.001), supporting Hypothesis 3a. As shown in Table 4, this interaction effect was significant at both moderator values, as the confidence intervals both times excluded zero [respectively, between −0.70 and −0.39 when head nurses’ autonomous motivation was low (−1 SD), and between −0.37 and −0.06 when head nurses’ autonomous motivation was high (+1 SD), at a 95% CI]. This means that head nurses’ autonomous motivation reduces the negative influence red tape has on their job satisfaction. This interaction is depicted in Figure 2, showing that while red tape decreases job satisfaction, head nurses’ autonomous motivation buffers this effect. Next, our analysis shows that head nurses’ autonomous motivation also reduces the negative influence of red tape on discretionary room (*B* = 0.20, *p* < 0.05), supporting Hypothesis 3b. As shown in Table 4, this effect was significant at both moderator values [respectively, between −0.75 and −0.33 when head nurses’ autonomous motivation was low (−1 SD), and between −0.48 and −0.04 when head nurses’ autonomous motivation was high (+1 SD), at a 95% CI]. Figure 3 gives a visual representation of this interaction, showing that while red tape decreases discretionary room, head nurses’ autonomous motivation buffers this effect. However, it is important to note that autonomous motivation is not capable of reversing the negative relationship between red tape and both discretionary room and job satisfaction.

**TABLE 4:** Results of the conditional direct and indirect effects for different levels of autonomous motivation.

	Effect	*SE*	LL 95% CI	UL 95% CI
**The conditional direct effect of red tape on job satisfaction**				
Low autonomous motivation (−1 *SD*)	−0.54	0.08	−0.6982	−0.3869
High autonomous motivation (+1 *SD*)	−0.22	0.08	−0.3706	−0.0609
**The conditional direct effect of red tape on discretionary room**				
Low autonomous motivation (−1 *SD*)	−0.54	0.11	−0.7458	−0.3260
High autonomous motivation (+1 *SD*)	−0.26	0.11	−0.4756	−0.0416
**The conditional indirect effect of red tape on job satisfaction (via discretionary room)**				
Low autonomous motivation (−1 *SD*)	−0.07	0.03	−0.1283	−0.0197
High autonomous motivation (+1 *SD*)	−0.03	0.02	−0.0745	−0.0016

*Participants’ age, gender, and span of control are included as control variables. Bootstrap sample size = 10,000.*

*N = 247; CI, confidence interval; LL, lower limit; UL, upper limit.*

Given the joint presence of mediation and moderation, we proceeded to test the presence of moderated mediation. The index of moderated mediation indicated that autonomous motivation moderates the mediated relationship between red tape and job satisfaction via discretionary room (Index of moderated mediation: 0.03; *SE*: 0.02; CI: 0.0041, 0.07), supporting Hypothesis 4. As can be seen in Table 4, the bootstrap results for the conditional indirect effect of red tape on job satisfaction, via discretionary room, indicated that this effect was significant at both moderator values [respectively, between −0.13 and −0.02 when head nurses’ autonomous motivation was low (−1 SD), and between −0.07 and −0.0016 when head nurses’ autonomous motivation was high (+1 SD), at a 95% CI].

## Discussion

By investigating the role of discretionary room and autonomous motivation in the relationship between head nurses’ red tape perceptions and their job satisfaction, this study did not only aim to provide additional knowledge regarding the underlying mechanisms in this relationship, but also to increase our understanding of how this suffering at work can be mitigated. Our analyses provide empirical evidence supporting each one of our hypotheses. Specifically, our results indicate that red tape undermines head nurses’ job satisfaction and that discretionary room acts as an underlying mechanism in this process. In addition, autonomous motivation mitigated the relationships between red tape and discretionary room and between red tape and job satisfaction. Both relations became weaker when head nurses reported higher levels of autonomous motivation. Lastly, autonomous motivation also affected the strength of the indirect relationship of red tape with job satisfaction through discretionary room. This mediated relationship became weaker when head nurses experienced more autonomous motivation.

### Theoretical Implications

This study makes multiple theoretical contributions. First of all, this study provides empirical evidence for a direct relationship between red tape and job satisfaction. More specifically, when head nurses perceive a lot of red tape in their job, they will experience lower levels of job satisfaction. This finding supports previous literature which already demonstrated a negative link between red tape and job satisfaction in other sectors and other contexts (e.g., Steijn and Van der Voet, 2019). By testing this relationship, we provide evidence that the same mechanisms also occur in a head nursing context. Hereby, we reveal that this relationship is also present in leadership positions, as previous research focused on examining this relationship among employees.

Secondly, as we collected our data amongst workers with managerial responsibilities, we were able to test whether red tape affects leaders’ managerial discretion. In this respect, our results suggest that red tape lowers head nurses’ discretionary room, which in turn lowers their job satisfaction. By focusing on this innovative mediator, we respond to calls for research to provide additional knowledge about the underlying mechanisms that explain how red tape affects employees’ attitudes and behaviors (e.g., Quratulain and Khan, 2015; George et al., 2021), and this in particular for employees that have a leadership position in the organization. This is an important contribution, as a better understanding of this relationship is crucial in order to stipulate well-founded recommendations regarding the red tape problem (George et al., 2021). Whereas discretionary room is an important advantage leaders can have compared to employees in non-supervisory positions, this advantage can be lowered when leaders experience red tape. This is all the more important in the face of leaders’ increasing job demands and shortages of leaders in many organizations’ leadership pipeline.

Lastly, this study reveals that autonomous motivation buffers the negative consequences of red tape. When head nurses are autonomously motivated, their discretionary room and their job satisfaction will be less negatively affected by the presence of red tape. Hereby, we provide empirical evidence supporting the JD-R model, which claims that personal resources can mitigate the negative consequences of hindering job demands (Bakker and Demerouti, 2018). Since autonomously motivated head nurses find their work interesting and spontaneously satisfying (Trépanier et al., 2013), their motivation helps them to cope with the presence of red tape. This finding contributes to research regarding the impact of personal resources when being confronted with red tape (Bakker and Demerouti, 2018). By identifying autonomous motivation as a key moderator, researchers are now one step closer to comprehending the complex relationship between red tape and job satisfaction. While previous literature focused on demonstrating the negative relation between red tape and job satisfaction (e.g., Steijn and Van der Voet, 2019), the present study found a way to buffer these negative consequences, and this specifically for leaders that are confronted with red tape in their job.

### Practical Implications

Occupational well-being, including job satisfaction, is receiving more and more attention since it became clear that it is an important determinant of performance and human functioning in general (Grant et al., 2007; Aziri, 2011; Bakker and Demerouti, 2018). For healthcare workers specifically, their occupational well-being in the form of job satisfaction does not only affect the organizations’ performance (e.g., Willem et al., 2007), it also risks affecting patients’ lives via quality and safety of patient care (e.g., Kvist et al., 2014). Ensuring healthcare workers’ job satisfaction should therefore be a top priority among practitioners. Therefore, it is important that we know which job characteristics affect healthcare workers’ job satisfaction, and well-being in general. Consequently, by providing evidence that both red tape and discretionary room affect healthcare leaders’ job satisfaction, this study is highly relevant for practitioners.

As our results suggest that red tape has a negative impact on head nurses, we give credence to initiatives that actively combat red tape, especially as we provide evidence that such measures can have far-reaching consequences (Blom et al., 2021; George et al., 2021). Consequently, policymakers and organizations need to thoroughly review the existing rules, regulations, and procedures in order to eliminate as much red tape as possible (Moynihan et al., 2012). This is of huge importance, not only to avoid dissatisfaction, but also because red tape hinders leaders’ discretionary room. We thus echo previous calls to practitioners that it is important that organizations hand sufficient freedom and authority to workers with managerial responsibilities (Knies and Leisink, 2014), and extend this call by adding that lowering red tape, wherever possible, can be one of the ways to tackle this problem.

Although red tape should be eliminated wherever possible, organizations will always be confronted with red tape, especially public and social profit organizations, as it might not be a viable option to remove all red tape (Walker and Brewer, 2009). Additionally, policymakers and organizations might not be able to remove externally imposed rules, regulations, and procedures, as they are often constrained in their ability to adapt the underlying regulations of workplace rules (Walker and Brewer, 2009; Moynihan et al., 2012). Fortunately, our findings also indicate that autonomously motivated head nurses are better equipped to handle red tape. Although autonomous motivation is not capable of reversing the negative relationship between red tape and both discretionary room and job satisfaction, it does mitigate red tape’s negative consequences. Consequently, as past research shows that employees’ and leaders’ level of autonomous motivation can be fostered by the organization (Fernet et al., 2021), workplace interventions with the purpose of increasing autonomous motivation would help individuals to deal with this hindering demand.

The level of autonomous motivation someone perceives depends on the satisfaction of the three basic psychological needs, namely, autonomy, competence, and relatedness (Ryan and Deci, 2000; Deci and Ryan, 2012; Amoura et al., 2013). People’s need for autonomy refers to their desire to make their own choices, to initiate their own behavior, and to behave in line with their own interests (Vandercammen et al., 2014). The need for competence refers to the need to feel effective and efficient in what you do (Ryan, 1995; Stockman et al., 2020). Lastly, the need for relatedness does not only refer to people’s necessity to feel connected to others, but also to feel accepted by them (Deci and Ryan, 2000; Vandercammen et al., 2014). Numerous prior studies have revealed that the satisfaction of these three basic psychological needs does indeed result in higher levels of autonomous motivation (Amoura et al., 2013; Vandercammen et al., 2014).

Previous literature has also investigated more specific ways on how organizations can foster the autonomous motivation of both their employees and leaders. First of all, past research shows that perceived organizational support leads to an increase in autonomous motivation (Gagné et al., 2010; Gillet et al., 2013; Chambel et al., 2015). Perceived organizational support refers to the degree to which people believe that their organization cares about their well-being and values their contributions (Eisenberger et al., 1986; Chambel et al., 2015). Consequently, investing in coaching activities, in formal and informal mentoring, and in creating a work environment conducive to communication, recognition, and information sharing will help to increase the autonomous motivation in an organization (Newman et al., 2012; Fernet et al., 2021). Additionally, leaders and directors can also foster subordinates’ autonomous motivation by showing autonomy-supportive behavior (e.g., Jungert et al., 2021). This can, for example, be done by providing positive feedback on performance, by providing a meaningful rationale for demands, by offering choices, by encouraging personal initiation, by acknowledging task difficulties, and by creating a work environment in which individuals have the freedom to determine how and when they perform their tasks (Deci and Ryan, 2000; Trépanier et al., 2013; Wang and Gagné, 2013; Vandercammen et al., 2014; Jungert et al., 2021).

Secondly, past research shows that it is important that employees and leaders internalize the organization’s goals in order to become autonomously motivated (Chen et al., 2020). This internalization happens when the organization clearly communicates what is important to them via their statement of purpose and their mission statement, and makes sure that their employees and leaders understand how they can contribute to achieving these objectives (Boswell, 2006; Chen et al., 2020). When individuals internalize organizational goals, they will feel less controlled in their work and more self-regulated, which will result in higher levels of autonomous motivation (Ryan, 1995; Chen et al., 2020). Moreover, and in line, past research also shows that strategic impact is positively related to autonomous motivation (De Cooman et al., 2013). Strategic impact refers to the effect someone’s personal work activities have on the accomplishment of general organizational objectives. In other words, by aligning individuals’ jobs with the organization’s general mission and objectives, organizations will improve the autonomous motivation of their employees and leaders.

Thirdly, past research also shows that transformational leadership and authentic leadership are both positively related to subordinates’ autonomous motivation (Bono and Judge, 2003; Wang and Gagné, 2013; Fateh et al., 2020). Transformational leaders autonomously motivate their subordinates by clearly communicating expectations, by articulating a vision, by gaining followers’ trust, and by spending time teaching and coaching their subordinates (Trépanier et al., 2013; Wang and Gagné, 2013). Authentic leaders, on the other hand, autonomously motivate their subordinates by supporting, encouraging, and validating their potential, by empowering them to find solutions on their own, by providing constructive and developmental feedback, and by letting them make their own decisions (Fateh et al., 2020). Consequently, encouraging such managerial practices amongst directors and leaders provides organizations with another promising avenue to promote subordinates’ autonomous motivation (Trépanier et al., 2013; Fateh et al., 2020).

Lastly, past literature shows that job complexity is positively related to autonomous motivation, as working on challenging tasks can help people to satisfy their need for competence and gives them the opportunity to demonstrate what they are capable of (De Cooman et al., 2013; Vandercammen et al., 2014; Fateh et al., 2020). Additionally, also competence and skill development practices, such as job training, job rotation, and learning and growth opportunities, are a very efficient way to foster autonomous motivation, as these practices convey the message to people working in the organization that their contributions are highly valued and that their employability is taken care of (Lee and Bruvold, 2003; Mustafa and Ali, 2019).

To conclude, when organizations focus on some of the above-mentioned practices, their leaders’ levels of autonomous motivation will increase. In turn, these higher levels of autonomous motivations will help them cope with the presence of red tape in their job, and consequently, their discretionary room and job satisfaction will be less negatively affected by the presence of red tape.

### Research Limitations and Future Directions

Despite the valuable findings of this study, several limitations are worth mentioning. First of all, future research should implement longitudinal or experimental designs to test the actual causality of our relations, for example through diary studies or experience sampling designs assessing red tape, as our results are based on cross-sectional data. Secondly, future research should consider using multi-source data, as our results are based on single-source perceptual data, which is susceptible to CSB (George and Pandey, 2017). Although our one-factor model suggests that influential CSB is absent, that CSB is not a universal inflator of the effect sizes of red tape (George et al., 2021), and that interaction effects cannot be an artifact of CSB (George and Pandey, 2017), it might still be interesting for future research to use multi-source data when investigating the impact of red tape. Next, future research would also benefit from taking red tape’s origin into account when investigating the buffering role of autonomous motivation, as past research revealed that red tape imposed by an organization itself is more detrimental than externally imposed red tape (George et al., 2021). Consequently, as the origin of red tape impacts its consequences, it would be interesting to investigate whether both internal and external red tape are buffered in a similar way. Lastly, future research could gain from investigating whether other personal resources, such as optimism and self-efficacy, are also capable to mitigate the negative consequences of red tape on leaders’ well-being, as this study only investigated the buffering role of autonomous motivation.

## Conclusion

Red tape is an increasing problem in many organizations around the world (e.g., Kaufmann et al., 2019). By unraveling the negative relationship between red tape and job satisfaction in a head nursing context, this study advances our understanding of the risks originating from the presence of red tape for leaders. More specifically, by revealing the mediating role of discretionary room in this relationship, this study provides additional knowledge regarding the underlying mechanisms that explain why workers with leadership positions are affected by red tape in a certain way. Given red tape’s negative consequences, it should be eliminated wherever possible. However, it might not be feasible for organizations to remove externally imposed regulations (Walker and Brewer, 2009; Moynihan et al., 2012). Consequently, leaders would benefit from the development of coping strategies to help them deal with the presence of red tape in their job (van Dorssen-Boog et al., 2021). Results of this study reveal that leaders’ autonomous motivation mitigates the negative relation between red tape and discretionary room and between red tape and job satisfaction. As autonomous motivation turns out to be an important protection mechanism against the negative consequences of red tape, organizations should put extra effort into stimulating the autonomous motivation of their leaders. When organizations make sure that their leaders’ job designs and work environments meet the need for autonomy, competence, and relatedness, leaders will become more autonomously motivated, which will buffer the negative impact of red tape.

## Data Availability Statement

The raw data supporting the conclusions of this article will be made available by the authors, without undue reservation.

## Ethics Statement

Ethical review and approval was not required for the study on human participants in accordance with the local legislation and institutional requirements. The participants provided their written informed consent to participate in this study.

## Author Contributions

JM: conceptualization, formal analysis, and writing. RB: conceptualization, data collection, formal analysis, and writing. MA and AD: conceptualization, data collection, and writing. All authors contributed to the article and approved the submitted version.

## Conflict of Interest

The authors declare that the research was conducted in the absence of any commercial or financial relationships that could be construed as a potential conflict of interest.

## Publisher’s Note

All claims expressed in this article are solely those of the authors and do not necessarily represent those of their affiliated organizations, or those of the publisher, the editors and the reviewers. Any product that may be evaluated in this article, or claim that may be made by its manufacturer, is not guaranteed or endorsed by the publisher.
